# Efficacy of an aid (gastric tube insertion guide) for oral insertion of a gastric tube: a randomized controlled trial

**DOI:** 10.1186/s40981-023-00597-w

**Published:** 2023-02-09

**Authors:** Shunsuke Saima, Naoi Tsurumachi, Takashi Asai, Yasuhisa Okuda

**Affiliations:** Department of Anesthesiology, Dokkyo Medical University Saitama Medical Center, Koshigaya, Saitama 343-8555 Japan

**Keywords:** Gastric tube insertion, Gastric tube guide, ERAS (enhanced recovery after surgery)

## Abstract

**Purpose:**

Insertion of a gastric tube orally may be more difficult than its insertion nasally, and thus, any aid to facilitate its insertion may be useful. Gastric tube insertion guide (Fuji Medical Corporation, Tokyo, Japan) has recently become commercially available. We felt that this device might be useful in facilitating oral insertion of a gastric tube, but there has been no formal study assessing its efficacy. The main aim of this study was to assess whether or not this “tube guide” would facilitate insertion of an orogastric tube.

**Methods:**

As a randomized controlled clinical study, we planned to study 40 patients, to assess the hypothesis that the success rate of insertion of an orogastric tube would be higher with the use of the “tube guide” than without. Patients were recruited when they were 20 years old, or older, and allocated randomly to one of two groups (20 people each group). In one group, the conventional “blind” insertion method was used and in the other group the “tube guide” insertion method.

**Results:**

The success rate was significantly higher for the “tube guide” method than the “blind” insertion method (*P* = 0.0012, 95% CI for difference: 23–67%).

**Conclusion:**

We have shown that the use of the gastric tube insertion guide® facilitates insertion of an orogastric tube.

## Introduction

Perioperative pulmonary aspiration can cause pneumonia and other serious complications [1, 2]. To minimize aspiration of gastric contents, a gastric tube may routinely be inserted either orally or nasally during general anesthesia or in the intensive care unit. One major problem with the use of a gastric tube is that its insertion may frequently be difficult, and factors causing its difficulty have not been clarified [3].

Recently, due to the idea of ERAS (enhanced recovery after surgery), it is recommended to remove a gastric tube at an early stage after operation [4, 5]. Therefore, if the gastric tube does not need to be left after surgery, it would be desirable to insert the tube orally instead of nasally, to prevent epistaxis and to reduce pain in the nasal cavity. Nevertheless, insertion of a gastric tube orally may be more difficult than its insertion nasally, and it is incomprehensible that any aid to facilitate its insertion may be useful.

**Fig. 1:**
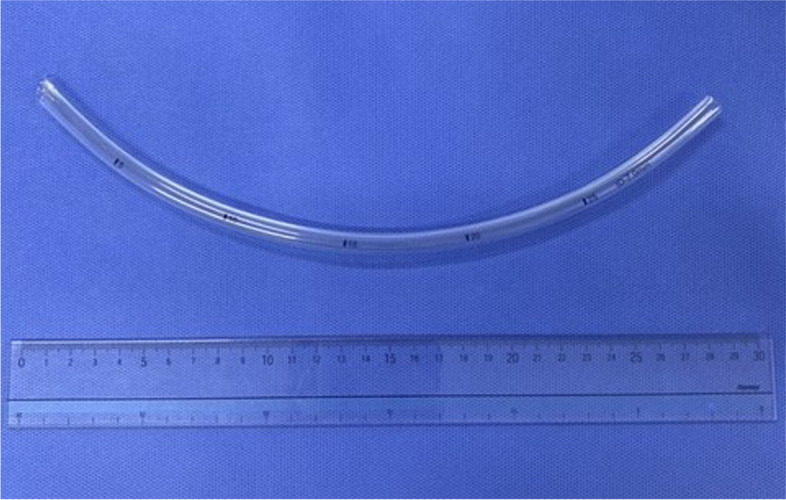
Gastric tube insertion guide (Fuji Medical)

Gastric tube insertion guide (Fuji Medical Corporation, Tokyo, Japan) (Fig. 1) has recently become commercially available. We have shown that this device facilitates insertion of a nasogastric tube [6]. We felt that this device might also be useful in facilitating oral insertion of a gastric tube, but there has been no formal study assessing its efficacy. The main aim of this study was to assess whether or not this “tube guide” would facilitate insertion of an orogastric tube.

## Methods

The research ethics committee of Dokkyo Medical University Saitama Medical Center approved the study (ID: 21041), and written informed consent was obtained from all the patients. The study was registered in a publicly accessible database before start of the study (jRCT1030210345).

As a randomized controlled clinical study, we planned to study 40 patients, to assess the hypothesis that the success rate of insertion of an orogastric tube would be higher with the use of the “tube guide” than without. Patients were recruited when they were 20 years old, or older, American Society of Anesthesiologists (ASA) physical status classification systems 1 or 2, scheduled for elective surgeries under general anesthesia, and in whom tracheal intubation was deemed necessary during anesthesia. Patients were excluded when they had esophageal varices or obstruction, history of head and neck trauma, radiation therapy, and surgery to the head and neck or to the upper gastrointestinal area. The patients with untreated coagulation abnormalities were also excluded.

Patients were allocated randomly to one of two groups (20 people each group). In one group, the conventional “blind” insertion method was used and in the other group the “tube guide” insertion method. Random allocation was made using a block randomization (blocks of 4), and each allocation was described in a card placed into a sealed opaque envelope. Insertion of a gastric tube was attempted by anesthesiologists who had used both methods for more than 10 times.

In the operation room, routine monitors, such as a blood pressure cuff, electrocardiogram, and pulse oximeter, were applied, an intravenous cannula was inserted, and a drip infusion was started. After 3 min of preoxygenation via a face mask (FiO_2_1.0 and flow rate: 6 L min^−1^), each anesthesiologist induced general anesthesia with propofol 2 mg kg^−1^ and fentanyl 2 μg kg^−1^, and neuromuscular blockade was achieved with rocuronium 0.6 mg kg^−1^. General anesthesia was maintained with sevoflurane in oxygen. When the anesthesiologist judged that neuromuscular blockade was adequate, the trachea was intubated. If the anesthesiologist judged that the depth of general anesthesia or neuromuscular blockade was insufficient, an additional dose of propofol, fentanyl, or rocuronium was injected. Each anesthesiologist opened an envelope and checked the card indicating the allocation and inserted a gastric tube orally, either by the conventional “blind” method or by the “tube guide” insertion method.

The “tube guide,” made of polyvinyl chloride, has a cylindrical hollow body structure with an inner diameter (ID) of 7.0 mm and allows passage of a gastric tube up to 18 Fr (6.0-mm outer diameter (OD)). The slit structure over the entire length of the tube facilitates the removal of the gastric tube after its successful insertion. The device can be used as an introducer for both an orogastric tube and a nasogastric tube. The guide is inserted orally to the esophagus. The tip of the gastric tube is inserted via the guide into the stomach. The guide is removed from the gastric tube.

Correct placement of a gastric tube was confirmed, by aspiration of gastric fluid or by auscultation of the epigastric region when injecting air through the gastric tube. We judged that insertion was failure, if it was not possible to confirm the correct placement of a gastric tube into the stomach within 180 s or if a gastric tube was inadvertently inserted into the trachea. The judgement of success or failure in all the patients was made by the same person.

Time to insertion, defined as the time from starting insertion of a gastric tube to confirmation of its correct insertion to the stomach, was measured by an independent person. If it was impossible to insert a gastric tube within 180 s, the other method (blind insertion for the “tube guide” group and the use of the “tube guide” for the “blind” group) was tried. If both methods failed, each anesthesiologist was allowed to choose the same or an alternative method (e.g., with the aid of a laryngoscope) of insertion of a gastric tube. If a gastric tube was inadvertently inserted to the trachea, insertion was judged failure, and the alternative insertion method was attempted. In addition, if serious complications, such as severe hypoxia or massive bleeding, occurred, we plan to terminate the study immediately and to start necessary treatment.

### Statistics analysis

Primary outcome measure was the success rate of insertion of an orogastric tube within 180 s. Secondary outcome measures included the time required for a successful insertion of a gastric tube, and the incidence of complications associated with insertion of a gastric tube.

Time for insertion is indicated as the median (range: interquartile range [IQR]), as the data were not normally distributed. Fisher’s exact test was used to compare the success rate of insertion of an orogastric tube, and the incidence of complications, between the groups. *P*-values < 0.05 were considered significant. The 95% confidence intervals for the difference in the success rate and in the incidence rate of complication were also calculated.

Our preliminary observation indicated that the success rate of insertion of an orogastric tube (without the use of an insertion aid) within 180 s was 50–60%, whereas it was 95–100% when an insertion aid was used. We considered that difference in success rate of 50% (55% versus 95%) would be clinically meaningful. To detect this, with a power of 80% and *P* = 0.05, 40 patients (20 patients per each group) would be required.

## Results

**Fig. 2:**
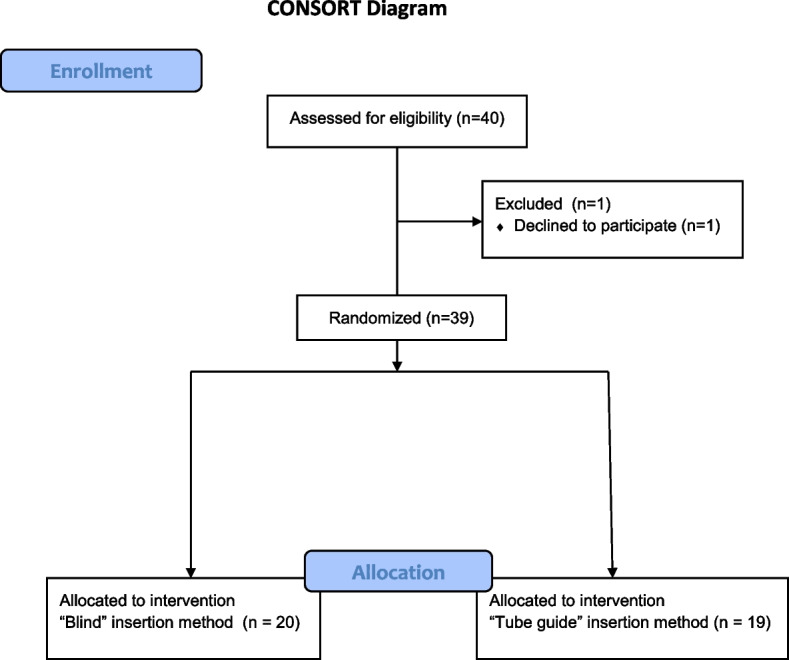
Consort diagram

We studied 39 patients, because one patient of group B withdrew agreement to participate (Fig. 2). Patient’s characteristics were similar between the groups (Table 1).

**Table 1:** Patients’ characteristics (mean (standard deviation))

	“Blind” insertion method	“Tube guide” insertion method
Age, years	65 (13)	64 (15)
Sex, M/F	7/13	8/11
BMI (kg/m^2^)	25 (4)	25 (5)
ASA physical status I/II	8/12	7/12

A gastric tube could be inserted within 180 s in all of 19 patients (100%) when the “tube guide” was used and in 11 of 20 patients (55%) when the “blind” method was used (Table 2). The success rate was significantly higher for the “tube guide” method than the “blind” insertion method (*P* = 0.0012, 95% CI for difference: 23–67%).

**Table 2:** Success rate and complication rate (%)

	“Blind” insertion method	“Tube guide” insertion method	*p*-values
Successful insertion	11/20 (55)	19/19 (100)	0.0012
Complications	1/20 (5)	7/19 (37)	0.0197

The median insertion time was 48 (*IQR*: 39.5–71.0) s for the “tube guide” insertion method and 68 (*IQR*: 54.3–80.3) s for the “blind” method. In 9 patients in the “blind” method group, insertion failed. In all of these patients, insertion was successful when the alternative method (“tube guide” method) was used.

Complications associated with insertion were observed in 7 of 19 patients (37%) with the “tube guide” method and 1 of 20 patients (5%) with the “blind” method. The incidence of complication was significantly higher with the “tube guide” method than with the “blind” method (*P* = 0.020, 95% CI for difference: 8–56%). In all of 7 patients of the “tube guide” method group, mild blood stain was detected to the tube guide (after its removal), whereas in 1 patient of the “blind” method group, the tube was inadvertently inserted to the trachea. No marked complications which required treatment occurred in any patients.

## Discussion

We have found that compared with the “blind” method, the use of the “tube guide” significantly increased the success rate of insertion of an orogastric tube. Although all the anesthesiologists had used both methods for more than 10 times, the experience with “tube guide” insertion method was far less than the “blind” method. Nevertheless, the success rate was significantly higher with the “tube guide” insertion method, and thus, our results suggest that insertion of a gastric tube using the “tube guide” is useful for oral insertion of a gastric tube.

Our results are similar to the results reported by Kriege et al. [7], who used a similar insertion aid (which is not available in Japan) (Gastric Tube Guide®, VBM Medizintechnik GmbH, Sulz am Neckar, Germany) to facilitate insertion of an orogastric tube. They reported that the incidence of complications was comparable between using the tube guide and a conventional blind insertion technique [7]. In contrast, we observed a higher incidence of complications (mainly mild mucosal bleeding) with the “tube guide” insertion method than with a “blind” method. One possible reason is that blood stain might not have been detected in patients of the “blind” method, since the tip of a gastric tube could not be confirmed immediately after insertion of a gastric tube. Nevertheless, caution is required when a gastric tube is inserted orally, with or without the use of a “tube guide”, as insertion of any tube into the esophagus has possibilities of esophageal rupture.

The videolaryngoscope is now widely available, so that the use of a videolaryngoscope during “tube guide” insertion is theoretically useful in confirming that the tube guide as well as a gastric tube is being correctly inserted to the esophageal inlet (and not the trachea) and in “opening” the esophageal inlet to facilitate insertion of the “tube guide.”

## Conclusion

We have shown that the use of the gastric tube insertion guide facilitates insertion of an orogastric tube, although it may increase minor complications (mainly mild mucosal bleeding).

## Data Availability

All the data are available from the authors.
